# Supplementary material for: The impact of the exercise on the social mentality of the Chinese people
Source: PLoS One. 2024 Sep 30;19(9):e0305972. doi: 10.1371/journal.pone.0305972 (PMC11441670; doi:10.1371/journal.pone.0305972)
Supplement: Attachment — Submitted filename: Response to Reviewers.docx [file pone.0305972.s002.docx]

Response to Reviewers

Dear Experts:

Greetings! Thank you very much for your valuable comments and suggestions on this article, we have benefited a lot.For each comment and suggestion, we have made careful deliberation and consideration, and carefully reviewed the relevant literature and information, and made corresponding modifications, and now the specific modifications are fed back as follows:

I**、Responses to modifications**

**1. Title: quite long; try to reduce to one sentence**

The author is very much in favor of your opinion. The title has been changed to "The impact of the exercise on the social mentality of the Chinese people". (line 1).

**2. Abstract: good**

No changes have been made here.
**3. Introduction: NOVELTY + results (better explanation)**

The author strongly agrees with your comments. Therefore, the introduction and literature review sections have been blended. The ideas presented in different studies are sorted out to provide arguments for this study. The argument is developed from the shift in people's need for sport from natural to social attributes, the positive and negative states presented by people's social mentality, and the impact of sport on social mentality. The effects and differences brought about by class, generation and space are also described. (lines 45-114).
**4. Literature review: for the part of social class theory, the authors must introduce newer literature (and not only Chinese). This part is very political and subjective.**

This has been integrated with the introductory section. Based on your comments, new literature has been introduced. The discussion of class is developed from different perspectives. Specifically: the relationship between people's trust in government and class; the dominance relationship between classes and social mentality; the impact of class-differentiated services of financial institutions on social mentality; and the relationship between the demand for sport and class mobility. (lines 76-92).
**5. Methodology: why the authors use only these indicators into the model? Present some theoretical explanations for these indicators**

The theoretical explanations for the selection of model indicators have been further expanded. See the indicator selection section in the research design. (lines 127-155).
**6. Model: Why in model 3/4/5, the authors introduce the variable class, which is very subjective and not used into analysis after that?**

The author is in deep agreement with you on this comment. The explanation of the model is enhanced with modifications. Located in the explanation and discussion of the statistical results, respectively. (lines 262-412, and lines 445-473).
**7. Model: the authors must introduce some tests for multicollinearity and endogeneity.**

We have gained a lot from this comment of yours. It motivates us to further standardize the use of statistical models and also further increase the reliability of the statistical results. Multicollinearity diagnostics added to the model setup section. Endogeneity tests were added to the statistical results section. Endogeneity tests were completed using the instrumental variables method. The original motion variable was replaced with an instrumental variable for whether or not the person participated in the motion. (lines 165-171, and lines 281-289).
**8. Model: the authors must introduce a robustness part.**

We have benefited greatly from this observation of yours. Accordingly, we used 2 methods for robustness testing, replacing the dependent variable and propensity score matching. It has been added in the statistical results section. The combination of social trust and social fairness as social mindfulness was used as a replacement for the dependent variable. Propensity score matching was performed to divide the experimental and control groups with the mean of the exercise variable, and the operation was completed using the control variable as the matching variable. (lines 290-297).
**9. Conclusions: at least 1 page**

Based on this comment of yours, we have expanded our conclusions. (lines 568-602).

II**、Explanation of other changes**

1、Some of the discussion in the text has been reorganized and corrected, and references and their serial numbers have been added and updated.

2、Reviewing the whole text, certain information details and language expressions in the text have been revised to make the presentation of views more accurate and concise.

III**、 the revised parts of the article have been marked in red font, please review them again!**

Finally, once again, I would like to express my heartfelt thanks to you for your valuable revisions and suggestions! I wish you happiness, well-being and success in your work!
